# Anti-inflammatory and nutritional improvement effects of dietary supplementation combined with fish oil in patients with epithelial cancer

**DOI:** 10.3892/ol.2022.13426

**Published:** 2022-07-12

**Authors:** Yumiko Shirai, Shunsuke Morita, Takashi Iwata, Hiroko Nakai, Mayu Yoshikawa, Kazuma Yoshida, Hiroshi Iwamoto, Kazuhiro Miyaji, Yoshinaga Okugawa, Chikao Miki, Koji Tanaka

**Affiliations:** 1 Department of Nutrition, Iga City General Hospital, Iga, Mie 518-0823, Japan; 2 Health Care and Nutritional Science Institute, Morinaga Milk Industry Co., Ltd., Zama, Kanagawa 252-8583, Japan; 3 Cancer Center, Aichi Medical University, Nagakute, Aichi 480-1195, Japan; 4 Department of Gastrointestinal and Pediatric Surgery, Mie University Graduate School of Medicine, Tsu, Mie 514-8507, Japan; 5 Heiikukai Medical Corporation, Tokyo 103-0002, Japan; 6 Department of Surgery, Iga City General Hospital, Iga, Mie 518-0823, Japan

**Keywords:** fish oil, eicosapentaenoic acids, cancer, nutrition, inflammation

## Abstract

The present study investigated the effects of dietary supplementation combined with fish oil containing relatively low levels of eicosapentaenoic acid (EPA) and docosahexaenoic acid (DHA) on the inflammatory and nutritional status of patients with epithelial cancer. Fish oil capsules (498 mg EPA and 213 mg DHA) and dietary supplements (100 kcal and 5 g protein) were administered for 8 weeks to 20 patients with cancer and inflammation [C-reactive protein (CRP) ≥0.30 mg/dl]. Blood EPA levels increased significantly after 4 and 8 weeks, while no significant differences were observed in log-transformed (log) CRP levels, which were the major inflammatory indices in these patients. A declining trend was observed at 8 weeks after excluding 2 patients with suspected infection (P=0.06). A significant increase was observed from week 0 to week 8 for log interleukin-6 (IL-6) levels. After excluding the 2 patients with suspected infection, no significant difference was observed when comparing week 0 to week 8 for log IL-6. No deterioration in albumin or pre-albumin levels was observed. These results suggest that although suppression of acute inflammation associated with infection is difficult, intake of relatively low EPA and DHA supplements may be effective for mild chronic inflammation in patients with epithelial cancer without infection. Large-scale randomized clinical trials are required to make the final decision regarding efficacy. The study was registered in the University Hospital Medical Information Network Clinical Trials Registry (UMIN-CTR; 06/07/2018, UMIN000033309).

## Introduction

Patients with cancer frequently experience nutritional disorders, with reports showing that these disorders occur in ~40% of cases (1). Nutritional disorders in patients with cancer affect the progression of the cancer and the treatment outcome, and are considered to worsen the cancer prognosis (2). Weight loss in patients with cancer is the result of reduced food intake due to a loss of appetite. However, cachexia secondary to inflammation may also be involved. Cancer cachexia promotes protein catabolism. This tends to reduce muscle mass and increase the likelihood of the development of sarcopenia (3). There have also been reports that cachexia promotes resting energy expenditure (REE) (4). Therefore, ensuring adequate food intake and controlling inflammation is essential in patients with cancer.

Inflammatory cytokines are elevated in cancer cachexia due to the immune response of the body and the production of cytokines by cancer cells (3). The severity of inflammation differs depending on the type and stage of the cancer (5,6), and the degree of invasiveness (7). Inflammatory cytokines, such as interleukin-6 (IL-6), promote catabolism of muscle proteins (8) and act on the central nervous system, leading to a reduced appetite (9). In addition to inflammatory cytokines, tumors produce proteolysis-inducing factor and lipid-mobilising factor, which promote the breakdown of skeletal muscle and adipose tissue (10,11). Increasing skeletal muscle mass by supplementation with nutrition alone is difficult; it is also essential to address the inflammatory state (12).

Under inflammatory conditions, C-reactive protein (CRP) synthesis predominates over albumin synthesis in the liver. As a consequence, CRP and albumin are considered to reflect the extent of the inflammation. The Glasgow Prognostic Score (GPS), advocated by Forrest *et al* (13), is a simple index involving CRP and albumin as prognostic predictors based on systemic inflammation evaluations. The GPS CRP cut-off value has been adjusted from 1.0 to 0.5 mg/dl for patients in Japan and this modified GPS (mGPS) has been used as the benchmark for cancer cachexia in our previous study (14).

Eicosapentaenoic acid (EPA) and docosahexaenoic acid (DHA) in fish oil exert anti-inflammatory effects. EPA is an n-3 fatty acid that antagonises the n-6 fatty acid arachidonic acid (ARA), and inhibits the production of inflammatory eicosanoids such as prostaglandin E_2_ (15). It has been reported that the EPA and DHA metabolites (resolvins) also have anti-inflammatory effects (16). Reports indicate that they inhibit inflammatory cytokine production and skeletal muscle catabolism (17,18). EPA and DHA are biosynthesized from the n-3 fatty acid α-linolenic acid; however, their conversion rate is limited. Therefore, the direct consumption of EPA and DHA is a more efficient means of increasing EPA and DHA levels in the body (19). A previous meta-analysis has reported the use of EPA and DHA to inhibit inflammation in patients with gastric cancer (20). While clinical studies have often used high amounts of fish oil to verify the anti-inflammatory effects of EPA and DHA, Mocellin *et al* (21) administered low amounts of fish oil (EPA 360 mg, DHA 240 mg) instead to patients with colorectal cancer during chemotherapy and reported a reduction in CRP. There is currently no consensus on the appropriate amount of EPA and DHA for patients with cancer and cachexia.

Cancer immuno-nutrition therapy using high-energy, high-protein products containing elevated levels of EPA has been used for anorexic patients with cancer cachexia to supplement their reduced food intake. In our previous study, it was reported that a combination of high-energy, high-protein dietary supplements with 2 g EPA not only increased skeletal muscle and lean body mass, but also improved the prognosis of patients with an mGPS of 1 or 2 who were administered prolonged chemotherapy (22). However, Aoyama *et al* (23) administered EPA-enriched dietary supplements to patients with cancer perioperatively and reported low patient compliance.

It is considered that high patient compliance could be maintained with low amounts of dietary supplements, in addition to fish oil capsules. The present study aimed to investigate the effects of combining relatively low amounts of EPA, DHA and dietary supplements on the inflammatory and nutritional status of cancer outpatients.

## Materials and methods

### Study design

This study was conducted between July 2018 and December 2019 at Iga City General Hospital (Iga, Japan) as a single-arm interventional study. The protocol was approved by the Ethics Review Committee of Iga City General Hospital (approval date: 4 June, 2018; approval no. 210). Written informed consent was obtained from all participants prior to the study. The study was conducted in accordance with the tenets of the Declaration of Helsinki (Fortaleza revision) and the Ethical Guidelines for Medical and Health Research Involving Human Subjects (Ministry of Education, Culture, Sports, Science and Technology and the Ministry of Health, Labour and Welfare Notice No. 3 of 2014, partially revised on 28 February 2017). The study was registered in the University Hospital Medical Information Network Clinical Trials Registry (UMIN-CTR; 06/07/2018, UMIN000033309).

### Subjects

The subjects were recruited from Iga City General Hospital. The inclusion criteria were an outpatient status, an age ≥20 years, a diagnosis of epithelial cancer, a CRP level ≥0.30 mg/dl, the capability for oral intake and at least 6 months of expected hospital visits as an outpatient. The exclusion criteria included food allergies (fish, milk, soya and gelatine) or possible food allergies, consumption of fish oil supplements, EPA/DHA products or steroid anti-inflammatories, participation in another clinical trial, the expectation of developing serious adverse events during the clinical trial period, being regarded as a difficult patient and being deemed unsuitable by the principal investigator. Patients using steroids for purposes other than anti-inflammatory agents such as antiemetics, were not excluded from the study. Surgical treatment, the use of EPA/DHA products, and the use of supplements containing EPA and DHA, and EPA and DHA fortified products was prohibited during the study period. The use of steroids as anti-inflammatory agents was prohibited.

### Dietary interventions

The subjects were provided with fish oil capsules (Umi no Genki EPA; Nippon Suisan Kaisha, Ltd.) and dietary supplements (Enjoy Small High-calorie Jelly; Morinaga Milk Industry Co., Ltd.) for 8 weeks, consisting of six fish oil capsules (498 mg EPA and 213 mg DHA) and one small high-calorie jelly (content, 40 g; energy, 100 kcal; protein, 5 g) once per day. The high-calorie jelly contained collagen peptides and whey proteins. Additionally, valine, leucine and isoleucine were added as branched-chain amino acids (BCAAs). The test food (fish oil capsules and small high-calorie jelly) was provided directly by the dietitian at the time of nutritional guidance. The time of the test food intake was not stipulated and subjects were asked to record their daily intake in a diary. The test food intake rate was calculated as [100× actual intake (number)/planned intake (number)]. Nutritional guidance was provided prior to the study (baseline) and at 4 and 8 weeks after commencement of the study. A 3-day dietary survey was conducted prior to the study period.

### Measurements

Body weight, body composition, REE and vital sign measurements (blood pressure, heart rate and body temperature), blood tests, and blood biochemical examinations were performed at baseline (week 0), and after 4 and 8 weeks of intake. CRP, IL-6, albumin and pre-albumin levels were measured as inflammation-related indicators. The inflammation-related indicators were measured by a clinical examination company (SRL, Inc.). Body composition was measured using a body composition analyzer (In Body S10; InBody Japan, Inc.), and REE was measured using an indirect calorimeter (MedGem; MP Japan Co., Ltd.).

### Statistical analysis

The target number of cases was set to 20, which is considered the maximum number of cases collected during the study. The test food intake rate is expressed as the mean ± standard error. Continuous variables were analyzed using linear mixed model analysis with a Tukey-Kramer post hoc test, and were expressed as the least-mean square values ± standard error. Additionally, individual data on inflammation-related indicators are shown as box plots. P<0.05 was used to indicate a statistically significant difference. The observation time point was defined as a fixed effect and the subject ID as a random effect. Logarithmic transformation was performed when continuous variables were non-normally distributed. Pearson's product-moment correlation coefficient was used to evaluate the inflammation-related indicators. All statistical analyses were performed using JMP13.2.1 software (SAS Institute, Inc.).

## Results

### Subject background characteristics

**Figure 1.**
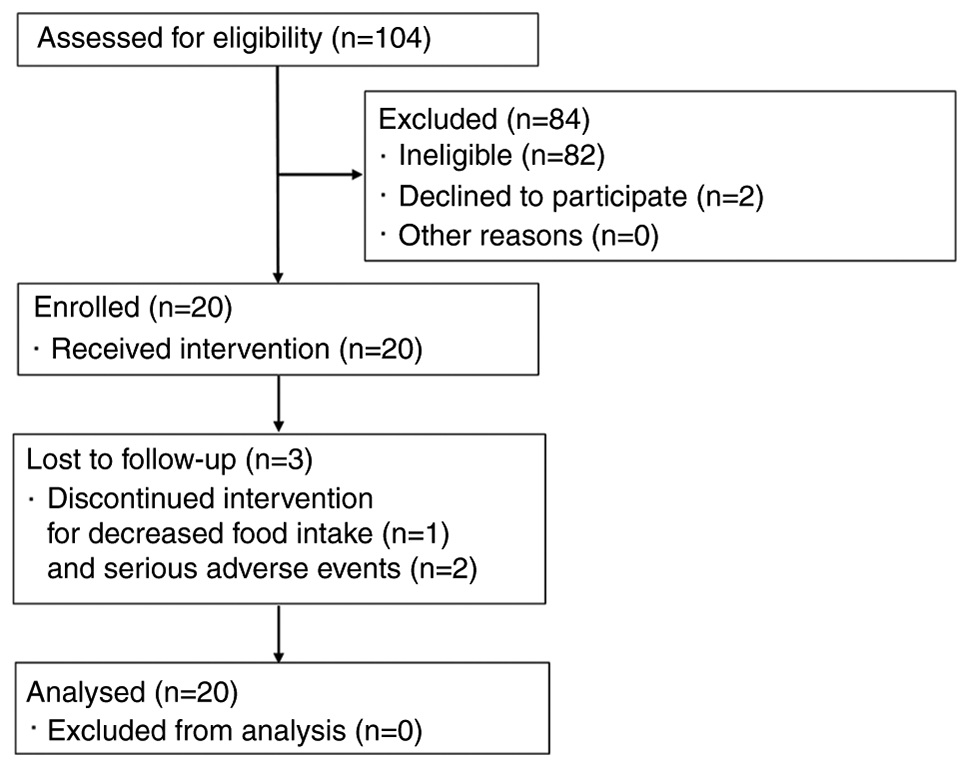
Flow diagram of patient selection.

A flow diagram of participants is shown in Fig. 1. Almost all ineligible patients had a CRP level <0.30 mg/dl. In total, 20 patients were enrolled in this study. Among them, 17 patients completed the trial, while 1 patient violated compliance requirements due to decreased food intake, and 2 patients withdrew due to serious adverse events. All 20 subjects were included in the efficacy analysis set. The diaries of 2 patients were not collected due to drop-out. These were considered compliance violations. A single patient violated the eligibility criteria due to the use of EPA preparations. In addition, 2 patients had findings suggestive of inflammation secondary to infection due to their sudden increase in CRP levels at week 8 and their good response to antibacterial drugs, and were excluded from the subgroup evaluation of inflammation-related indicators. In 1 of these excluded cases, the observed adverse events included lower extremity peripheral neuropathy, high cholesterol, cracked skin on the hands, reduced magnesium, chills, fever, elevated CRP level, slightly elevated white blood cell count, and reduced pre-albumin level. In the other case, adverse events included malaise, peripheral neuropathy, cheilitis, stomatitis, elevated CRP level and dysphagia. A causal relationship between such adverse events and test food was ruled out.

The baseline characteristics of the subjects and the results of the 3-day dietary survey are shown in Table I. The median age of the subjects was 72 years (range, 44–94 years). The types of cancer included epithelial colorectal (n=16), stomach (n=2), lung (n=1) and pancreatic (n=1) cancer. In total, 17 subjects underwent chemotherapy at baseline. Test food intake rates of fish oil capsules and dietary supplements were 97.3±0.9% (n=18) and 95.6±1.1% (n=18), respectively.

### Body composition, vital signs and REE

The changes in body weight and body composition are shown in Table II. No significant changes were observed in any of the measurements. The changes in vital signs (blood pressure, heart rate and body temperature) and REE are shown in Table III. No significant changes were observed in any of the measurements.

### Clinical test items

**Figure 2.**
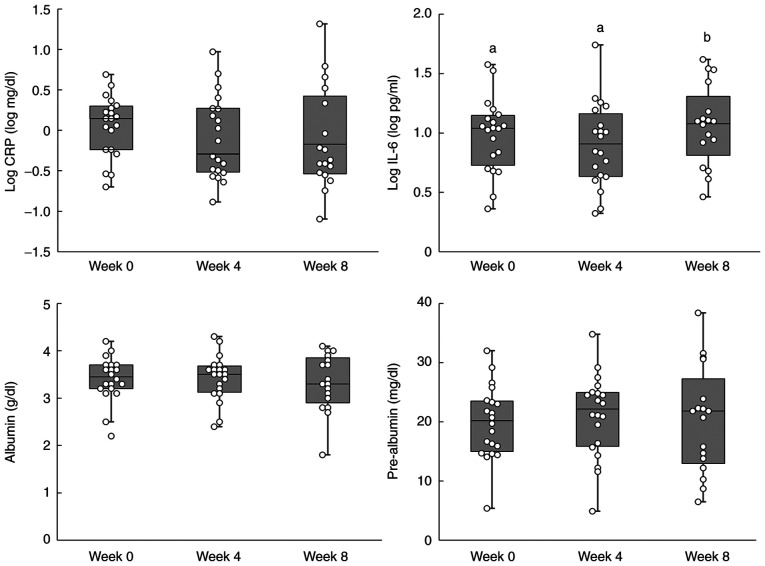
Box plots for inflammation-related indicators. Inflammation-related indicators were recorded at weeks 0, 4 and 8. The number of subjects was 20, 20 and 17, respectively. The boxplot shows the median and quartiles. Different superscript letters indicate a significant difference between groups (P<0.05), while the same letters indicate no significant difference (P>0.05) (Tukey-Kramer post hoc test). CRP, C-reactive protein; IL-6, interleukin 6.

**Figure 3.**
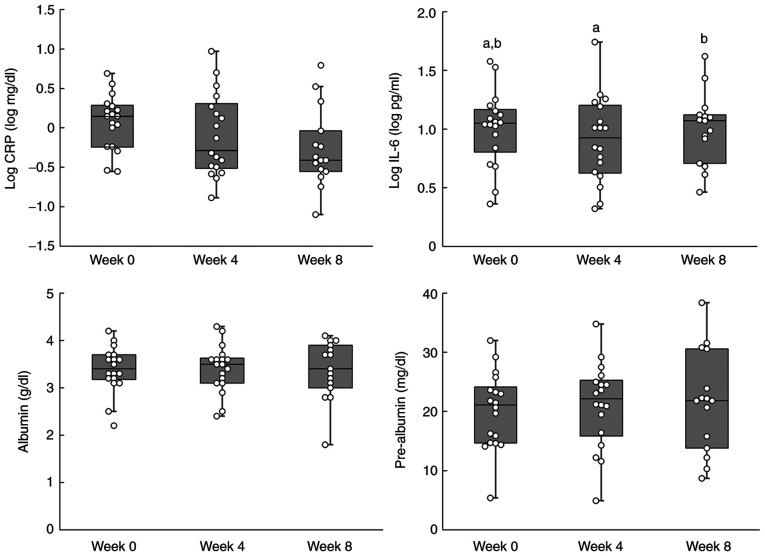
Boxplots for inflammation-related indicators (subgroup analysis). Inflammation-related indicators were recorded at weeks 0, 4 and 8, excluding those for 2 patients with suspected infections. The number of subjects was 18, 18 and 15, respectively. The boxplot shows the median and quartiles. Different superscript letters indicate a significant difference between groups (P<0.05), while the same letters indicate no significant difference (P>0.05) (Tukey-Kramer post hoc test). CRP, C-reactive protein; IL-6, interleukin 6.

The results for the inflammation-related indicators are shown in Table IV, and the individual data are shown in Fig. 2. No significant differences were observed in log-transformed (log) CRP levels. A significant increase in log IL-6 levels was observed after 8 weeks. Furthermore, no significant differences were observed in albumin and pre-albumin levels. The results of the subgroup analysis, which excluded the 2 cases with suspected acute inflammation secondary to infection, are shown in Table V. Furthermore, individual data for the sub-group analysis are shown in Fig. 3. The log CRP level showed a significant trend in the subgroup analysis, which was performed using a linear mixed model. A decreasing trend was also observed in the subgroup analysis performed using the Tukey-Kramer post-hoc test (week 0 vs. week 8; P=0.0632). However, post-hoc analyses are not routinely performed when no significant differences are observed in data. A significant difference in log IL-6 levels was not observed between weeks 0 and 8, although a significant increase was observed between weeks 4 and 8. The blood tests and biochemical examination results are shown in Table VI. With regard to urea nitrogen levels, significant changes were observed in urea nitrogen levels between weeks 0 and 4, although no significant differences were observed between weeks 0 and 8. The correlation coefficients of inflammation-related indicators are listed in Table VII. A positive correlation was observed between log CRP and log IL-6, and between albumin and pre-albumin, while a negative correlation was observed between log CRP and albumin, and between log CRP and pre-albumin.

### Serum fatty acid composition

**Figure 4.**
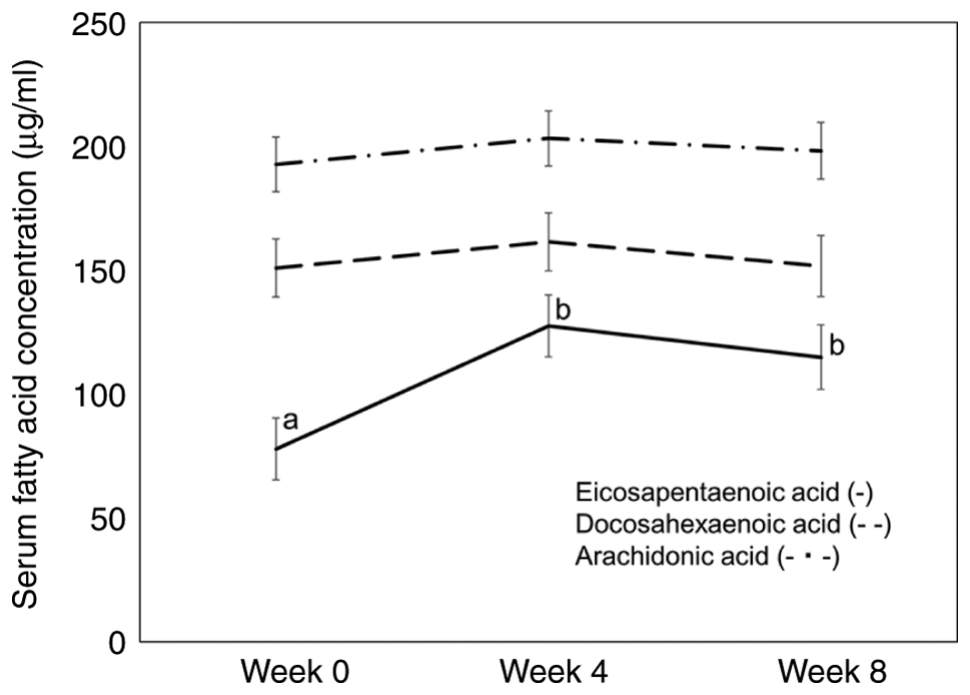
Changes in serum fatty acid concentrations. Least-mean square value ± standard error values are presented. Different superscript letters indicate a significant difference between groups (P<0.05), while the same letters indicate no significant difference (P>0.05) (Tukey-Kramer post hoc test

The main changes in fatty acid composition (%) are shown in Table VIII. Serum EPA, docosapentaenoic acid, and EPA/ARA ratios significantly increased at weeks 4 and 8 compared with week 0. Serum oleic acid level significantly decreased at week 4 compared with that at week 0 and 8. Serum EPA, DHA and ARA concentrations are shown in Fig. 4. Serum EPA concentrations significantly increased in weeks 4 and 8 compared with those in week 0.

## Discussion

No significant changes in log CRP, albumin and pre-albumin levels were observed for patients following intake of the test food for 8 weeks. Log IL-6 levels significantly increased between week 0 and week 8. The test food intake rates remained high and the general nutritional status of the patients was well maintained. CRP levels in the subgroup analysis showed a downward trend at week 8 using linear mixed-model analysis with a Tukey-Kramer post hoc test. In addition, a significant difference from week 0 was not observed at week 8 for log IL-6 levels. These findings indicate that although suppression of acute inflammation secondary to infection is difficult with the study intake amounts of EPA and DHA, they may exert suppressive effects on mild chronic inflammation in patients with epithelial cancer without infection.

It has been suggested that the anti-inflammatory effects of n-3 fatty acids present in EPA and DHA in patients with colorectal cancer may differ depending on the factors involved in the perioperative period and during chemotherapy. A meta-analysis reported a reduction in IL-6 inflammatory cytokines and an increase in albumin in the overall analysis. The intake of a relatively small amount of EPA and DHA during chemotherapy decreased CRP levels, but not IL-6 levels (24). By contrast, another meta-analysis involving patients with gastric cancer, in which 8 out of the 9 included trials involved surgical patients, found no changes in CRP levels but a reduction in IL-6 levels (20).

IL-6 is known to increase markedly in the perioperative period due to invasive surgery (7). Intake of high-dose fish oil is considered essential to inhibit IL-6 production. By contrast, chemotherapy causes inflammation due to the immune response of the body to cancer and the inflammatory substances produced by the cancer cells. If cancer cells can be controlled with chemotherapy, then IL-6 production is assumed to be relatively suppressed, resulting in the persistence of a chronic mild inflammatory state. However, even when inflammation is mild, chronic persistence may result in muscle mass reduction. Therefore, it is preferable to suppress inflammation completely. In these instances, even a relatively small amount of fish oil was assumed to exert an anti-inflammatory effect.

Cancer patients are at a high risk of infection due to their reduced immunity during both the perioperative and chemotherapy periods. CRP levels are markedly elevated during infection (25,26). Consequently, when using CRP as an inflammation indicator, it is essential to distinguish primary inflammation from that secondary to infection. This lack of distinction is considered to be one of the reasons for the difficulty in verifying the inflammatory suppression effects of fish oil. As in the present clinical study, the present study had some cases with suspected acute inflammation secondary to either an infection or acute exacerbation of cancer. Only 2 cases were excluded in the subgroup analysis due to their good response to antibacterial drugs. However, it cannot be denied that the infection might have affected CRP levels in the other cases. In the present study, a downward trend in log CRP was observed after excluding the 2 cases in which infection was strongly suspected. This suggested that the intake of fish oil may work to suppress mild chronic inflammation; however, the difference was not statistically significant. In this study, the suppression of acute inflammation caused by infection was considered challenging. Hence, further research is required to investigate the effects of fish oil on the suppression of inflammation triggered by cancer cells during chemotherapy.

A number of the subjects from the present study consumed fish daily; hence, the serum levels of EPA and DHA were relatively high. Particularly, the levels of DHA were high, and they did not increase with the intake of fish oil capsules. Fish oil capsules have a high EPA content, and serum EPA levels were significantly elevated. Both EPA and DHA are known to have anti-inflammatory effects, although in this study, it was assumed that the effects were primarily elicited by EPA. Plasma DHA levels were maintained at a relatively high state from baseline, which may reflect a constant anti-inflammatory effect. Antagonism of ARA, which promotes inflammation, is known to be an anti-inflammatory action of EPA (15). Therefore, the EPA/ARA and ARA/EPA ratios are considered useful inflammatory indicators (27). Although no significant changes in ARA were observed in this study, the EPA/ARA ratio was found to be significantly elevated. EPA and DHA may also exert anti-inflammatory actions through metabolites such as resolvins that also have anti-inflammatory actions (16). These actions are considered to lead to a reduction in inflammatory cytokines and CRP levels. A study by Mocellin *et al* (21) found an increase in plasma EPA and DHA levels, and a decrease in ARA levels, when lower amounts of fish oil (360 mg EPA and 240 mg DHA) were ingested compared with the value observed in the present study. Given that the baseline plasma EPA level was low and the ARA level was high, it is assumed that the diets of their subjects contained mainly n-6 fatty acids and that the elevated plasma DHA and reduced ARA levels may have also contributed to the reduction of CRP. Hamazaki *et al* (28) reported that a relatively low amount of fish oil (600 mg EPA and 260 mg DHA) decreased serum triglyceride and remnant-like particle-cholesterol in normolipidemic and hypertriglyceridemic subjects. In the study, the participants habitually consumed fish, and the intake of DHA was estimated at 670–830 mg/day. In addition, the EPA levels in red blood cells increased, but the DHA levels did not. The relatively small amount of fish oil (~500 mg EPA) may be physiologically meaningful for mild metabolic disorders, including inflammation, in Japanese patients who habitually consume fish.

In a comparison analysis of 3 studies focusing on reducing triglycerides levels by plasma EPA concentrations, the baseline values increased from 7.9, 28.1 and 63.6 µg/ml to 108.9, 123.8 and 185.0 µg/ml, respectively with the administration of a 2-g EPA preparation (29–32). In the present study, as a number of the subjects consumed fish daily, baseline plasma EPA concentrations were high, and DHA intake levels, including intake from fish oil capsules, were also high. This suggests that high plasma EPA concentrations could be maintained even with a relatively small amount of EPA supplementation. The high daily intake of fish was also considered to be one of the contributing factors to the downward trend in CRP.

In the present study, a decline in log CRP at week 8 was observed in all the patients, except for the 2 patients with suspected infection, although no changes in log IL-6 were observed at week 8 compared with week 0. A decline in CRP levels was also reported by Mocellin *et al* (21), with no changes in inflammatory cytokines such as TNF-α. Silva Jde *et al* (33) also found that the intake of small amounts of fish oil (EPA 360 mg, DHA 240 mg) produced no differences in the levels of inflammatory cytokines, such as IL-6, when compared to a group without fish oil intake. However, there was a decrease in the trends of CRP in the fish oil-supplemented group compared with those in the control group. IL-6 induces CRP production in the liver. A previous report suggested that EPA may also inhibit CRP production in the liver through IL-6 (34). This suggests that the reduction of CRP levels due to EPA may also be involved in the downstream inhibition of IL-6.

Chronic inflammation in patients with cancer is one of the causes of cachexia, and EPA-enriched dietary supplements are utilized to improve the nutritional status of patients. A previous study reported that intake of high-energy, high-protein dietary supplements with a high EPA content may be useful for inhibiting caloric intake-dependent weight loss in patients with inoperable pancreatic cancer (35). By contrast, a study on perioperative conditions found that although compliance was good, no changes in body weight or improvement in the nutritional status were observed with nutritional supplementation (36). High-dose dietary supplements also present a problem in terms of a tendency for low compliance (23). Maintaining a high continuation rate of test food intake is important in immuno-nutrition therapy. The present study combined the use of fish oil capsules with low-content, high-energy products, both of which have high compliance rates. However, no significant changes in the nutritional status of the patients were observed. Supplement intake (calories and protein) might not be sufficient for weight and muscle increase. The main purpose of this study was to evaluate the levels of cancer-related anti-inflammatory markers. An improvement in nutritional status was expected to accompany an anti-inflammatory response. Therefore, very small and high-calorie commercial supplements were chosen to avoid the impact of food intake. The dietary intake of the study subjects was good, and the body mass index (BMI) was maintained at ~22 kg/m^2^. Mocellin *et al* (21) also reported high baseline BMI and no weight loss, even in the control group. By contrast, Silva Jde *et al* (33) reported weight loss in the control group and a significant increase in the fish oil-supplemented group compared with that of the control group. If the dietary intake is adequate, there should be no weight loss even in mildly inflammatory states. This makes verifying effects on the improvement in nutritional status difficult. In the present study, no significant changes in muscle mass were demonstrated. However, mild inflammatory conditions are known to cause sarcopenia. Therefore, a longitudinal clinical study is essential to verify this finding.

Albumin is also used to evaluate the nutritional status. The mean albumin value in the present study was slightly lower than the mGPS reference standard (3.5 mg/dl). The synthesis of albumin is known to decrease under inflammatory conditions and a negative correlation was found between log CRP and albumin levels in this study. Considering the changes in dietary intake and body weight, this suggests that the decrease in albumin is due more to the effects of inflammation, rather than due to energy or protein deficiency. A declining trend in log CRP was seen by excluding the 2 subjects strongly suspected of having an infection, although no increase in albumin levels was demonstrated.

The limitations of the present study include the single-arm interventional design and the small sample size. The inflammatory status is often increased in patients with cancer during chemotherapy and their nutritional status may worsen. In this study, there was no deterioration in albumin and pre-albumin levels. However, as this was a single-arm interventional study, it was not possible to rule out the possibility that the deterioration of the nutritional status may have been inhibited. Mocellin *et al* (21) reported that albumin levels did not change in the fish oil intake group but declined in the control group. Therefore, the possibility that the progression of the inflammatory condition may have been inhibited cannot be ruled out. Large-scale randomized clinical trials are required to make the final decision regarding efficacy.

The present study excluded perioperative patients and targeted outpatients. The study also did not restrict the inclusion of patients undergoing chemotherapy, as the main reason for an outpatient visit among patients with cancer is for chemotherapy. There was also no restriction on the type or stage of the cancer. We predicted that an anti-inflammatory effect would be expected regardless of the type of cancer in cases of mild chronic inflammation. As a result, the majority of the subjects were patients undergoing chemotherapy for colorectal cancer. The study subjects had good dietary intake and BMI values, and the significance of adding dietary supplements was not obtained from this study. It may be better to restrict the evaluation of dietary supplementation to patients with a poor nutritional status. Patients with cancer cachexia are prone to malnutrition due to reduced dietary intake associated with anorexia and due to their increased inflammatory status. Dietary supplements, small amounts of high EPA content for energy and protein supplementation are considered useful for improving malnutrition in patients with cancer cachexia. However, there is no established evidence. Large-scale, randomized clinical trials are required to investigate the appropriate amounts of EPA and DHA, as well as the nutritional intake of cancer patients.

In conclusion, the present results suggest that although suppressing acute inflammation associated with infection is challenging, the intake of relatively small amounts of EPA and DHA may be effective for mild chronic inflammation in patients with epithelial cancer without infection. Large-scale randomized clinical trials are required to make the final decision regarding efficacy. The participants in this study had a relatively good nutritional status, although no improvement was observed. Further studies are required to determine the appropriate levels of EPA, DHA and dietary intake to inhibit inflammation and improve malnutrition in patients with cancer.

## Figures and Tables

**Table I.** Subject baseline characteristics.

Characteristic	Value
Age, years	69.6±2.6
Sex (male:female), n	17:3
Height, cm	165.2±1.6
Stage (III:IV), n	3:17
Energy intake, kcal/day	1909±108.2
Protein intake, g/day	72.3±4.7
Fat intake, g/day	66.0±6.2
Carbohydrate intake, g/day	239±10
EPA intake, mg/day	320±94
DHA intake, mg/day	598±143
Vitamin D intake, µg/day	9.0±1.4
Zinc intake, mg/day	8.1±0.6

Data are presented as mean ± standard error unless stated otherwise (n=20). EPA, eicosapentaenoic acid; DHA, docosahexaenoic acid.

**Table II.** Changes in body weight and body composition.

Test element	Week 0 (n=20)	Week 4 (n=18)	Week 8 (n=17)	P-value
Body weight, kg	61.5±2.6	62.1±2.7	62.9±2.7	0.1367
BMI, kg/m2	22.6±1.0	22.8±1.0	23.1±1.0	0.1566
Muscle mass, kg	43.9±1.2	43.7±1.2	44.6±1.2	0.4374
Body fat percentage	23.1±2.0	24.2±2.0	23.8±2.0	0.2003
Body water, l	34.3±0.9	34.2±0.9	34.9±0.9	0.3804
ECW/TBW	0.391±0.003	0.391±0.003	0.395±0.003	0.2527
TBW/FFM	73.8±0.1	73.8±0.1	73.8±0.1	0.7479
Skeletal muscle mass, kg	25.3±0.8	25.1±0.8	25.5±0.8	0.7369
Protein amounts, kg	9.0±0.3	9.0±0.3	9.1±0.3	0.6595
Bone mineral content, kg	2.55±0.09	2.58±0.09	2.69±0.09	0.1173
Body fat, kg	15.0±2.1	15.8±2.1	15.7±2.1	0.0629
Intracellular water, l	20.9±0.6	20.8±0.6	21.1±0.6	0.7540
Extracellular water, l	13.4±0.4	13.4±0.4	13.8±0.4	0.1109

Data are presented as the least-mean square value ± standard error. P-values were generated using linear mixed-model analysis. BMI, body mass index; ECW, extracellular water; TBW, total body water; FFM, fat-free mass.

**Table III.** Changes in vital signs and REE.

Measured outcomes	Week 0 (n=20)	Week 4 (n=19)	Week 8 (n=17)	P-value
Systolic blood pressure, mmHg	129±3	135±3	129±3	0.1670
Diastolic blood pressure, mmHg	80±2	77±2	77±2	0.4093
Heart rate, bpm	87±3	87±3	85±3	0.8706
Body temperature, °C	36.4±0.1	36.4±0.1	36.4±0.1	0.9705
REE, kcala	1278.8±55.5	1293.2±60.0	1345.7±61.9	0.5708

aWeek 0, n=17; week 4, n=14; and week 8, n=13. Data are presented as the least-mean square value ± standard error. P-values were generated using linear mixed-model analysis. REE, resting energy expenditure.

**Table IV.** Inflammation-related indicators.

Test element	Week 0 (n=20)	Week 4 (n=20)	Week 8 (n=17)	P-value
Log CRP, log mg/dl	0.05±0.12 (1.12)	−0.10±0.12 (0.79)	−0.04±0.12 (0.91)	0.4338
Log IL-6, log pg/dl	0.98±0.08a (9.5)	0.90±0.08a (7.9)	1.13±0.08b (13.5)	0.0072
Albumin, g/dl	3.41±0.12	3.43±0.12	3.26±0.12	0.1117
Pre-albumin, mg/dl	19.89±1.68	21.05±1.68	19.65±1.74	0.5205

Data are presented as the least-mean square value ± standard error. P-values were generated using linear mixed-model analysis. Different superscript letters within a row indicate a significant difference between groups (P<0.05), while the same letters indicate no significant difference (P>0.05) (Tukey-Kramer post hoc test). Values in brackets show the antilogarithm conversion from the logarithm of the least-mean-square value. CRP, C-reactive protein; IL-6, interleukin-6.

**Table V.** Inflammation-related indicators in subgroup analysis.

Test element	Week 0 (n=18)	Week 4 (n=18)	Week 8 (n=15)	P-value
Log CRP, log mg/dl	0.078±0.111 (1.20)	−0.093±0.111 (0.81)	−0.173±0.117 (0.67)	0.0637
Log IL-6, log pg/dl	1.01±0.08a,b (10.2)	0.91±0.08a (8.1)	1.08±0.08b (12.0)	0.0324
Albumin, g/dl	3.39±0.13	3.41±0.13	3.30±0.13	0.4156
Pre-albumin, mg/dl	20.14±1.79	21.13±1.79	20.81±1.85	0.7344

Data are presented as the least-mean square value ± standard error. P-values were generated using linear mixed-model analysis. Different superscript letters within a row indicate a significant difference between groups (P<0.05), while the same letters indicate no significant difference (P>0.05) (Tukey-Kramer post hoc test). Values in brackets show the antilogarithm conversion from the logarithm of the least-mean-square value. CRP, C-reactive protein; IL-6, interleukin-6.

**Table VI.** Blood tests and biochemical examinations.

Test element	Week 0 (n=20)	Week 4 (n=20)	Week 8 (n=17)	P-value
White blood cell count, cells ×103/µl	5.5±0.5	5.8±0.5	6.5±0.5	0.2694
Red blood cell count, cells ×106/µl	4.06±0.13	4.09±0.13	3.94±0.13	0.2471
Haemoglobin, g/dl	11.9±0.4	12.2±0.4	11.9±0.4	0.6102
Haematocrit, %	36.2±1.1	37.0±1.1	35.9±1.1	0.3331
Platelet count, cells ×103/µl	214±20	214±20	208±21	0.8996
Total protein, g/dl	6.4±0.1	6.4±0.1	6.1±0.1	0.1170
Log AST, log IU/l	1.48±0.04	1.51±0.04	1.49±0.05	0.6123
	(30)	(32)	(31)	
Log ALT, log IU/l	1.39±0.05	1.38±0.05	1.39±0.06	0.9942
	(25)	(24)	(25)	
Urea nitrogen, mg/dl	14.5±1.5a	17.6±1.5b	15.9±1.5a,b	0.0346
Log creatinine, log mg/dl	−0.08±0.02	−0.07±0.02	−0.08±0.03	0.7352
	(0.83)	(0.85)	(0.83)	
Log triglycerides, log mg/dlc	2.13±0.05	2.09±0.05	2.14±0.05	0.4444
	(135)	(123)	(138)	
Total cholesterol, mg/dlc	212±13	231±13	214±13	0.1786
LDL cholesterol, mg/dl)	132±11	146±11	136±11	0.1118
HDL cholesterol, mg/dl	51±3	57±3	54±4	0.0897
25-OHVD, ng/ml	13.2±1.2	13.4±1.2	12.4±1.3	0.1948
Total carnitine, µmol/l	45.5±4.8	50.8±4.8	48.1±4.9	0.3456
Free carnitine, µmol/l	36.1±3.8	39.5±3.8	38.4±3.9	0.4614
Acyl carnitine, µmol/l	9.5±1.2	11.0±1.2	9.3±1.3	0.4554

cWeek 0, n=13; week 4, n=13; and week 8, n=12. Data are presented as the least-mean square value ± standard error. P-values were generated using linear mixed-model analysis. Different superscript letters within a row indicate a significant difference between groups (P<0.05), while the same letters indicate no significant difference (P>0.05) (Tukey-Kramer post hoc test). Values in brackets show the antilogarithm conversion from the logarithm of the least-mean-square value. ALT, alanine aminotransferase; AST, aspartate aminotransferase; LDL, low-density lipoprotein; HDL, high-density lipoprotein; 25-OHVD, 25-hydroxyvitamin D.

**Table VII.** Correlation between inflammation-related indicators.

Variable	vs. variable	Correlation coefficient	P-value
Log CRP	Log IL-6	0.7323	0.0008
Log CRP	Alb	−0.5585	0.0198
Log CRP	PreAlb	−0.7117	0.0014
Log IL-6	Alb	−0.3852	0.1268
Log IL-6	PreAlb	−0.2952	0.2499
Alb	PreAlb	0.6359	0.0061

CRP, C-reactive protein; Alb, albumin; IL-6, interleukin 6.

**Table VIII.** Serum fatty acid composition.

Test element	Week 0 (n=20)	Week 4 (n=20)	Week 8 (n=17)	P-value
Myristic acid, %	0.97±0.12	0.85±0.12	0.90±0.12	0.3862
Palmitic acid, %	23.52±0.44	23.00±0.44	23.48±0.45	0.2569
Palmitoleic acid, %	2.11±0.17	1.98±0.17	2.07±0.17	0.4703
Stearic acid, %	7.17±0.15	7.02±0.15	7.25±0.16	0.1033
Oleic acid, %	22.33±0.70a	19.80±0.70b	21.35±0.73a	0.0004
Linoleic acid, %	25.87±0.80	27.15±0.80	25.69±0.84	0.1458
Linolenic acid, %	0.82±0.05	0.79±0.06	0.74±0.06	0.3038
Dihome-γ-linolenic acid, %	1.10±0.07	1.01±0.07	0.99±0.07	0.0992
ARA, %	5.42±0.36	5.74±0.36	5.69±0.37	0.4062
EPA, %	2.19±0.37a	3.71±0.37b	3.24±0.38b	<0.0001
Docosapentaenoic acid, %	0.58±0.04a	0.74±0.04b	0.72±0.04b	0.0002
DHA, %	4.21±0.34	4.61±0.34	4.30±0.35	0.2500
Other, %	3.71±0.12	3.62±0.12	3.57±0.12	0.1167
EPA/ARA	0.39±0.06a	0.66±0.06b	0.61±0.06b	<0.0001

Data are presented as the least-mean square value ± standard error. P-values were generated using linear mixed-model analysis. Different superscript letters within a row indicate a significant difference between groups (P<0.05), while the same letters indicate no significant difference (P>0.05) (Tukey-Kramer post hoc test). EPA, eicosapentaenoic acid; ARA, arachidonic acid; DHA, docosahexaenoic acid.

## Data Availability

The datasets used and/or analyzed during the current study are available from the corresponding author on reasonable request.
